# Resistance of *Biomphalaria alexandrina* to *Schistosoma mansoni* and *Bulinus truncatus* to *Schistosoma haematobium* Correlates with Unsaturated Fatty Acid Levels in the Snail Soft Tissue

**DOI:** 10.1155/2020/8852243

**Published:** 2020-11-01

**Authors:** Marian Elias, Rasha S. Hanafi, Samia El-Bardicy, Ebtisam A. Hafez, Rashika El Ridi

**Affiliations:** ^1^Department of Malacology, Theodore Bilharz Research Institute, Giza 12411, Egypt; ^2^Faculty of Science, Cairo University, Giza 12613, Egypt; ^3^Department of Pharmaceutical Chemistry, Faculty of Pharmacy and Biotechnology, German University in Cairo, New Cairo City, Cairo 11835, Egypt

## Abstract

Only a fraction of the *Biomphalaria* and *Bulinus* snail community shows patent infection with schistosomes despite continuous exposure to the parasite, indicating that a substantial proportion of snails may resist infection. Accordingly, exterminating the schistosome intermediate snail hosts in transmission foci in habitats that may extend to kilometres is cost-prohibitive and damaging to the ecological equilibrium and quality of water and may be superfluous. It may be more cost effective with risk less ecological damage to focus on discovering the parameters governing snail susceptibility and resistance to schistosome infection. Therefore, laboratory bred *Biomphalaria alexandrina* and *Bulinus truncatus* snails were exposed to miracidia of laboratory-maintained *Schistosoma mansoni* and *S. haematobium*, respectively. Snails were examined for presence or lack of infection association with soft tissue and hemolymph content of proteins, cholesterol, and triglycerides, evaluated using standard biochemical techniques and palmitic, oleic, linoleic, and arachidonic acid, assayed by ultraperformance liquid chromatography-tandem mass spectrometry. Successful schistosome infection of *B. alexandrina* and *B. truncatus* consistently and reproducibly correlated with snails showing highly significant (up to *P* < 0.0001) decrease in soft tissue and hemolymph content of the monounsaturated fatty acid, oleic acid, and the polyunsaturated fatty acids, linoleic, and arachidonic acids as compared to naïve snails. Snails that resisted twice infection had soft tissue content of oleic, linoleic, and arachidonic acid similar to naïve counterparts. High levels of soft tissue and hemolymph oleic, linoleic, and arachidonic acid content appear to interfere with schistosome development in snails. Diet manipulation directed to eliciting excessive increase of polyunsaturated fatty acids in snails may protect them from infection and interrupt disease transmission in a simple and effective manner.

## 1. Introduction

Schistosomiasis caused by trematode worms of the genus *Schistosoma* is a debilitating disease associated with significant morbidity and mortality. Three members, *Schistosoma mansoni*, *Schistosoma haematobium*, and *Schistosoma japonicum*, are responsible for the great majority of approximately 300 million human infections and 800 million, principally children, at risk of the infection [1, 2]. Schistosomiasis is also termed snail fever as schistosomes' life cycle involves asexual reproduction in a compatible, fresh water snail whereby infection with a single miracidium results in production of thousands of infective cercariae [3]. Incidence and prevalence of schistosomiasis reflect the distribution of the freshwater intermediate host snail [1–3]. Snails *Oncomelania* density, rate of infection, and zones range, size, and proximity to human habitation were reported to be responsible for the high prevalence of schistosomiasis japonicum in the Philippines and southern China [4, 5]. In the Middle East and sub-Saharan Africa, *Oncomelania* spp. and schistosomiasis japonicum are not found while the widespread incidence of schistosomiasis mansoni and schistosomiasis haematobium is a function of the habitat range and distribution of susceptible snail species of the genus *Biomphalaria* and *Bulinus*, respectively [6–10]. In South America and the Caribbean, the distribution of *Biomphalaria* spp. (*B. glabrata*, *B. straminea*, *B*. *tenagophila*) is closely associated with the occurrence of schistosomiasis mansoni [11]. Accordingly, the use of chemical [12–15] and plant-derived [16–18] molluscicides was advocated for schistosomiasis control and transmission elimination, yet, was not strongly encouraged due to probable toxicity to aquaculture flora and fauna.

Habitats of the intermediate snail hosts in transmission foci within water bodies, along rivers, lakes, and ponds may extend to kilometres. Attempts at snail extermination with molluscicides may raise threats to the ecological equilibrium and quality of water for drinking and irrigation, vegetation, and animal life and could well be unnecessary. That is because only a fraction of the snail community shows patent infection despite continuous exposure to the parasite, indicating that a substantial proportion of snails may resist infection [7, 19–23]. Discovering the snail resistance underlying immunogenic mechanisms may well guide procedures and methods towards eliminating schistosomiasis [21].

The success of schistosome snail infection was reported to be a function of matching between the host and parasite phenotype [21, 24, 25]. The “matching hypothesis” claims that gene-encoded diversification and polymorphisms of the effector/antieffector systems and antigens and receptors of the parasite and the host lead to phenotype matching or mismatching between the schistosome and the snail, resulting in infection success and failure, respectively [21, 24, 25]. Mechanisms which correlate with resistance include too hemocyte abundance, rate of migration from hemolymph to soft tissues, higher cupper/zinc superoxide dismutase activity and capacity to produce hydrogen peroxide (H_2_O_2_), and more potent ability to encapsulate and kill the invading and developing schistosome larvae [26–33].

Additionally, resistance to infection was reported to reflect parasite-induced unfavorable biochemical changes in the intrasnail environment [21, 34, 35]. Resistance to infection of the water rat with *S. mansoni* was associated with increase in arachidonic acid content in the liver [36]. Susceptibility and resistance of the laboratory mouse, *Mus musculus*, and rat, *Rattus norvegicus*, to *S. mansoni* directly correlated with low and high serum arachidonic acid levels, respectively [37]. Poor development of *S. mansoni* in T and B lymphocytes-deficient mice appeared to be a function of elevated arachidonic acid levels in serum [38]. The antischistosome activity of sclareol and its heck-coupled derivatives was attributed to the drug-mediated enhancement of arachidonic acid metabolism in the target worms [39]. Additionally, arachidonic acid was shown to be an effective schistosomicide of *S. mansoni* and *S. haematobium in vitro* and *in vivo* in mice and hamsters, respectively [40–42]. *Schistosoma haematobium* larvae and adult worms were consistently more susceptible to the unsaturated fatty acids, namely, arachidonic acid and parasiticidal impact *in vitro* [40–44] and *in vivo* [40–42]. Arachidonic acid was also reported to be an effective therapy for schistosomiasis mansoni light infection in children [45, 46]. Recently, arachidonic acid was shown to function as a principal schistosomicidal and parasite ovocidal mediator of the adjuvant-free, cysteine peptidase-based schistosomiasis vaccine [47].

These findings together urged examining the role of intramolluscan polyunsaturated fatty acid (PUFA) levels in the susceptibility/resistance of the laboratory-bred snails *Biomphalaria alexandrina* and *Bulinus truncatus* to infection with the Egyptian laboratory strains of *S. mansoni* and *S. haematobium*, respectively. Accordingly, protein content and levels of cholesterol, total neutral triglycerides, palmitic, oleic, linoleic, and arachidonic acid levels were evaluated in the hemolymph and soft tissues of algae or lettuce-fed *B. alexandrina* and *B. truncatus* snails. The data obtained following infection with the parasites are the first, to our knowledge, to examine the effect of diet on snails' innate resistance to parasites and fully supported the influence of soft tissue PUFAs, notably arachidonic acid, levels in the snail resistance to infection.

## 2. Materials and Methods

### 2.1. Snails and Parasites

*B. alexandrina* and *B. truncatus* snails were obtained from the Schistosome Biological Supply Centre–Theodore Bilharz Research Institute (SBSC-TBRI), Giza, Egypt, where they were bred for several years under standardized laboratory conditions. Dechlorinated tap water has a pH of 7.1 ± 0.1, total dissolved solids of 340 ± 10 mg/l, and dissolved oxygen of 6.65 ± 1 mg/l. Temperature is maintained at 26 ± 1°C and light exposure at 12 h fluorescent light and 12 h dark. Miracidia of *S. mansoni* and *S. haematobium* were used for snail infection immediately after hatching of the eggs isolated from the liver of 7- and 12-week infected hamsters, respectively, as described [48].

### 2.2. Infection

Adult *B. alexandrina* and *B. truncatus* were both divided into two major groups each, one lettuce fed and the other algae fed. Three groups each of thirty algae-fed snails and six groups each of thirty lettuce-fed snails were exposed overnight, on an individual snail basis, to freshly hatched miracidia (8-10 miracidia/*B. alexandrina* snail and 15-20 miracidia/*B. truncatus* snail), under direct light at temperature of 26 ± 1°C. The snails were then maintained in standard aquaria with clean dechlorinated water (10 snails/l). After the prepatent period (30 days for *S. mansoni* and 40 days for *S. haematobium*), each of *B. alexandrina* (85.5% and 66.6% for algae and lettuce-fed snails, respectively) and *B. truncatus* (46.6% and 43.3% for algae and lettuce-fed snails, respectively) snail that was detected to shed cercariae was interchangeably labelled susceptible or infected. Snails that were unable to shed cercariae were subjected to an additional cycle of infection as described above, and if proven to lack cercarial shedding at the end of the second prepatent period (0% of algae-fed *B. alexandrina* and *B*. *truncatus* and 7.7% and 16.6% % of lettuce-fed *B. alexandrina* and *B*. *truncatus*, respectively), they were labelled resistant to infection.

### 2.3. Sample Collection

The head-foot of the snail was exposed (by cracking the shell around it without disturbing the animal inside) and pierced with a fine needle a short distance below the mouth. The exuded hemolymph was collected as quickly as was possible before it could have been contaminated by secretions from the mucous gland. Collected hemolymph for each individual snail was centrifuged at 2,000 × *g* for 3 min, the pellet was discarded, and the supernatant was divided into the two Eppendorf tubes each containing 50 *μ*l of hemolymph, one for the biochemical assays and the other for the ultraperformance liquid chromatography-tandem mass spectrometry (UPLC-MS/MS) analysis. Soft tissue samples were collected by crushing the snails individually (after hemolymph collection) and divided into two Eppendorf tubes each containing 50 mg of soft tissue, one for the biochemical assays and the other for the UPLC-MS/MS analysis.

### 2.4. Protein, Cholesterol, and Total Neutral Triglyceride Assays

Hemocyte-free hemolymph was used. As for the soft tissue, it was homogenized in 500 *μ*l ultrapure, pyrogen free water containing 0.05% (*w*/*v*) sodium dodecyl sulfate (SDS), centrifuged at 2,000 × *g*, and the clear supernatant was used. Total protein content (5 replicates per group) was analysed using Bradford Protein Assay kit (Bio-Rad Laboratories Inc., Hercules, CA, USA), following the manufacturer instructions. Samples were added in duplicate wells of 96-well plates (10 *μ*l/well), incubated at 25 ± 1°C for 15 min with occasional light shaking and absorbance read at 630 nm (ELx808, BioTek, Bad Friedrichshall, Germany). Total protein content was expressed as mg/ml.

Cholesterol and neutral triglyceride levels were evaluated in 5 replicates per each group using colorimetric assay kits (Cholesterol assay kit, Chronolab Systems, Barcelona, Spain, and Triglycerides assay kit, Reactivos GPL, Barcelona, Spain, respectively). In 96-well plates, 200 *μ*l of reagent was added to the wells along with 2 *μ*l standard, 2 *μ*l hemolymph, or 2 *μ*l soft tissue extract samples adjusted to contain 3 mg/ml protein, i.e., 6 *μ*g/well. The plates were incubated at 25 ± 1°C for 15 min with occasional light shaking and absorbance read at 540 nm (ELx808). Cholesterol and triglyceride content was expressed as mg/mg protein.

### 2.5. Fatty Acid Extraction and Assay

Fatty acids were extracted from samples of hemolymph (50 *μ*l) and soft tissue (50 mg) on an individual snail basis, following the Folch method [49] with an added extraction step to ensure the purity of the resulted fatty acid extract. Butylated hydroxytoluene (BHT) 1 : 30 *w*/*v* was added to the chloroform/methanol mixture as an antioxidant to prevent any oxidation of the extracted fatty acids [50]. All samples and chemicals were kept on ice at all times. Extracted fatty acids were reconstituted in 100 *μ*l hexane to chloroform (3 : 1)/HBT and stored at -20°C until assayed by UPLC-MS/MS. Samples were allowed to completely dry from hexane to chloroform (3 : 1) solvent mixture on ice in a fume hood and reconstituted in 500 *μ*l HPLC grade methanol and membrane (0.22 *μ*m syringe filter, Membrane Solutions, Shanghai, China) filtered in glass vials before injection in UPLC-MS/MS. Liquid chromatography was done using an UPLC system (ACQUITY UPLC; Waters Corporation, Milford, MA, USA) on Acquity BEH C18 Column (1.7 *μ*m, 2.1 mm × 50 mm; Waters Corporation) and analysed with a Triple quadrupole MS/MS (Xevo TQ-S micro, Waters). Elution was isocratic, where mobile phase was 65% of 5 mM ammonium acetate in acetonitrile and 35% 5 mM ammonium acetate in isopropanol. Flow rate was 0.2 ml/min; column temperature was 40°C. Data were collected using MRM (Multiple Reaction Monitoring) in negative ESI (Electrospray Ionization) mode. The capillary voltage was 4 kV, cone voltage 62 V, radio frequency (RF) lens voltage 2.5 V, and source temperature 200°C. Nitrogen was used as the desolvation and cone gas at a flow rate of 650 l/hr, while argon was used as the collision gas at a pressure of approximately 3.67 × 10^−3^ mbar. System operation and data acquisition were controlled using the MassLynx® 4.1 software (Waters Corporation). The method was validated according to ICH guidelines of bioanalytical method validation where the range of linearity was evaluated by testing different dilutions of the four fatty acids in HPLC grade methanol (palmitic acid (Cat# P0500, Sigma, St. Louis, MO, USA), oleic acid (Cat# O1008, Sigma, USA), linoleic acid (Cat# L1376, Sigma, USA), and arachidonic acid (Cat# A3611, Sigma, USA)) ranging from 5 ng/ml to 20,000 ng/ml, where the linear dynamic range was found to be from 50 ng/ml to 20,000 ng/ml and limit of detection (LOD) and limit of quantitation (LOQ) were 3 ng/ml and 10 ng/ml, respectively. The reproducibility, accuracy, and precision were calculated by running a selected array of 6 standard concentrations as quality control samples (50 ng/ml, 500 ng/ml, 2000 ng/ml, 6000 ng/ml, 10,000 ng/ml, and 18,000 ng/ml) in between the snail samples' runs and evaluating the intra- and interday data. For calibration curves, a serial dilution (0.05-20 *μ*g/ml in HPLC grade methanol) of the four fatty acids was used. Linear regression analysis was performed to correlate peak area ratios of fatty acids (peak area fatty acid/peak area of internal standard (docosahexaenoic acid, Sigma) to fatty acid concentrations (Supplementary Table 1A-D; Supplemental Figure 1A-E)).

### 2.6. Histology and Light Microscopy Studies

Twelve days after miracidium infection, snails were initially relaxed with methanol crystals. Each snail was carefully crushed between two microscope slides, and the broken shell pieces pulled away from the soft tissue with care. The columellar muscle was successfully separated from the shell, and the snail soft tissue extracted intact. Snails' soft tissue was stretched to their full length on a glass slide, fixed with a few droplets of Bouin's fixative, and transferred to Bouin in a sterile Eppendorf for at least 24 hours. Bouin-treated soft tissue samples were dehydrated in gradually increased concentrations of ethanol, cleared in xylol, and embedded in paraffin blocks. Using a microtome, 5 *μ*m sections from the paraffin block were cut and stained with haematoxylin-eosin dye. Haematoxylin-eosin-stained 5 *μ*m sections were examined under light microscopy for histological condition of larval trematodes, as categorized by Borges et al. [51], with 10x lens. Sections of interest were photographed using 40x lens.

### 2.7. Statistical Analysis

One-way ANOVA tests were used to analyse the statistical significance of differences between all groups of the same snail species and considered significant at *P* < 0.05. Calculations were performed using PRISM® 5.01 (GraphPad, San Diego, CA, USA).

## 3. Results

### 3.1. Biomphalaria Alexandrina

#### 3.1.1. Effect of Diet

**Figure 1:**
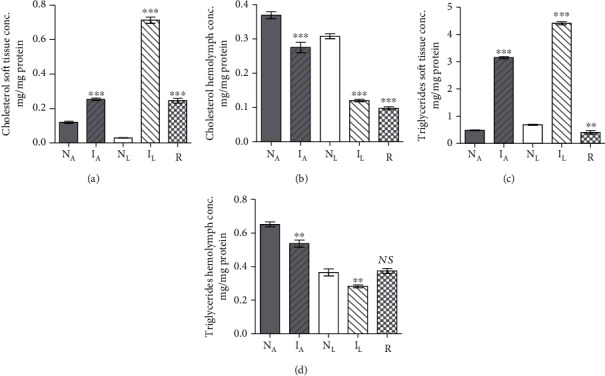
Each column represents mean *B. alexandrina* soft tissue cholesterol (a), hemolymph cholesterol (b), soft tissue triglycerides (c), and hemolymph triglycerides (d) content for five naïve-algae fed (N_A_), infected-algae fed (I_A_), naïve-lettuce fed (N_L_), infected-lettuce fed (I_L_), resistant snails (R), and vertical bars the SE about the mean. Statistical differences between naïve and infected or resistant same diet-fed snails are shown; ^∗∗∗^*P* < 0.001, ^∗∗^0.001 < *P* < 0.005. NS: not significant.

**Figure 2:**
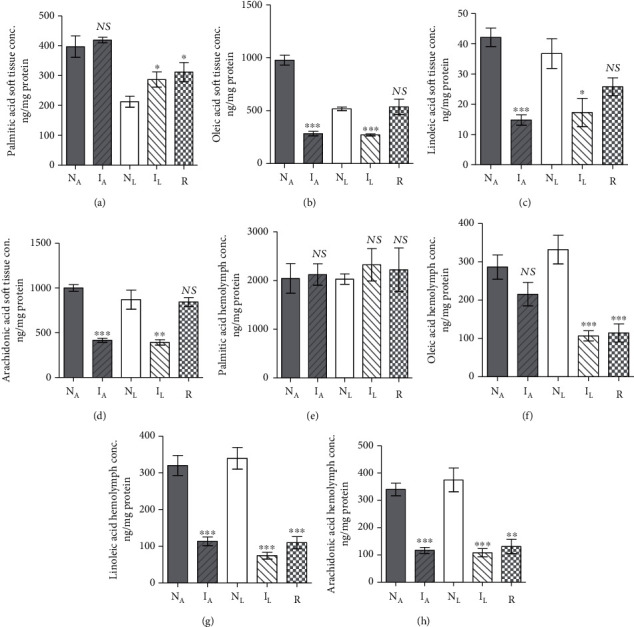
Each column represents mean *B. alexandrina* soft tissue and hemolymph fatty acid level content. Each column represents mean soft tissue palmitic (a), oleic (b), linoleic (c), and arachidonic (d) acid and hemolymph palmitic (e), oleic (f), linoleic (g), and arachidonic (h) acid content for five *B. truncatus* snails, and vertical bars the SE about the mean. N_A_: naïve-algae fed; I_A_: infected-algae fed; N_L_: naïve-lettuce fed; I_L_: infected-lettuce fed; R: resistant snails, and vertical bars the SE about the mean. Statistical differences between naïve and susceptible or resistant same diet-fed snails are shown; ^∗∗^0.001 < *P* < 0.005; ^∗∗∗^*P* < 0.001. NS: not significant.

Two experiments were performed in tandem. In each, five healthy *B. alexandrina* snails maintained in parallel in identical conditions, but on lettuce and algae diet, were assessed for soft tissue and hemolymph protein and lipid content on an individual snail basis. The statistical analyses of results revealed that the diet difference had no significant impact on soft tissue and limited effect on hemolymph protein content (Supplementary Tables 2, 3). Lettuce diet elicited decrease in soft tissue cholesterol, hemolymph triglycerides (Figures 1(a)–1(d)), and soft tissue palmitic and oleic acid (Figures 2(a) and 2(b)) levels as compared to algae-fed snails, perhaps providing an explanation for detection of resistant snails only among those on lettuce diet. Otherwise, there was no differential impact of algae and lettuce diet on fatty acid levels in soft tissue and hemolymph (Figures 2(a)–2(h)).

#### 3.1.2. Effect of Infection in Algae-Fed Snails

In two independent experiments, five *B. alexandrina* snails maintained on algae diet were subjected to infection with *S. mansoni* miracidia and assayed 30 days later for soft tissue and hemolymph protein and lipids content in parallel with entirely naïve snails. Infection failed to alter the snail soft tissue and hemolymph protein (Supplementary Tables 2, 3) or palmitic acid (Figures 2(a) and 2(e)) content. The most salient changes 30 days *S*. *mansoni* infection induced regarding cholesterol, and triglyceride parameters were highly significant (*P* < 0.005) increase in soft tissue accompanied with decline in hemolymph (Figures 1(a)–1(d)). Of note, infection resulted in highly significant (*P* < 0.0001) decrease in soft tissue oleic, linoleic, and arachidonic acid levels and in hemolymph linoleic and arachidonic acid content when compared with naïve snails (Figure 2).

#### 3.1.3. Effect of Infection in Lettuce-Fed Snails

Snail infected with *S. mansoni* miracidia for 30 days did not greatly differ from their naïve counterparts regarding the protein content of soft tissue or hemolymph (Supplementary Tables 2, 3). Like algae-fed snails, 30 days *S*. *mansoni* infection induced cholesterol and triglycerides highly significant (*P* < 0.005) level increase in soft tissue and decline in hemolymph (Figures 1(a)–1(d)). Of note, 30 days *S. mansoni* infection in lettuce-fed snails led to some increase in palmitic acid content (Figures 2(a) and 2(e)), while led to highly significant (*P* < 0.001) decline in oleic, linoleic, and arachidonic acid level soft tissue and hemolymph, when compared with naïve snails (Figures 2(a)–2(h)).

#### 3.1.4. Resistance versus Susceptibility Parameters

Laboratory bred naïve snails were maintained on algae or lettuce diet and unexposed to any infection. A series of two sets of five snails were exposed twice to infection with 5-10 of *S. mansoni* miracidia, with one-month interval. The snails, which shed cercariae either after the first or the second experimental infection, were labelled infected and considered susceptible. Resistant snails were detected uniquely among lettuce-fed hosts. All three groups (naïve, infected, and resistant) were assessed for soft tissue and hemolymph protein and lipids content on an individual snail basis. Snail resistance to infection with *S. mansoni* miracidia was associated with minor decrease in soft tissue and hemolymph protein content (Supplementary Tables 2, 3), and variable changes in cholesterol, triglycerides (Figures 1(a)–1(d)), and palmitic acid (Figures 2(a) and 2(e)) levels. Highly significant (*P* < 0.0001) decline in hemolymph oleic, linoleic, and arachidonic acid content was recorded when compared to lettuce-fed naïve snails (Figures 2(f)–2(h)). In contrast to susceptible group, resistant snails failed to decrease their soft tissue oleic (Figure 2(b)), linoleic (Figure 2(c)), and arachidonic (Figure 2(d)) acid content; mg PUFA/mg protein values were nearly similar to those of naïve snails. Resistance of snails to support *S. mansoni* development remarkably correlated with the inability to decrease the soft tissue content of PUFAs to levels that are not lethal to the larvae.

#### 3.1.5. Histological Examination

**Figure 3:**
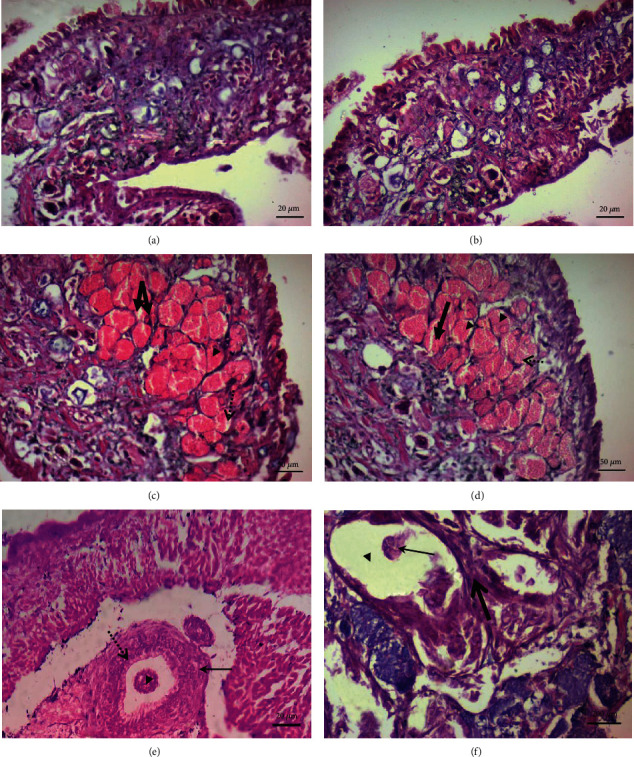
Histological transverse sections of *B. alexandrina* snails showing cephalopodal tissue. (a, b) are of naïve snails maintained on algae (a) and lettuce (b). (c, d) are of infected snails maintained on algae and lettuce, respectively, showing live *S. mansoni* mother sporocysts with normal development pattern; some of them contain single sporocyst cluster (thin arrow), and others contain multiple sporocyst clusters (thick arrow). (e, f) are of snails that resisted infection. Cephalopodal tissue is seen showing dead sporocyst (thin arrow) surrounded by granuloma-like structure consisting of layers of flattened hemocytes followed by concentric layer of fibroblasts (thick arrow) (e). The mantle collar region shows the sporocysts losing their identical cluster-like shape and presence of germinal cells lacking nucleoli. The size of the vacant space (thin arrow) is wider than in normal sporocysts. Note, presence of fibroblast lamination (thick arrow) surrounding the sporocysts' tegument (f).

Haematoxylin sections of *B. alexandrina* snail soft tissues at 12 days postinfection displayed limited hemocytes involvement in naïve (Figures 3(a) and 3(b)), infected (Figures 3(c) and 3(d)), resistant (Figures 3(e) and 3(f)), algae- (Figures 3(a) and 3(c)), or lettuce- (Figures 3(b) and 3(d)–3(f)) fed snails, despite that the correct timing was selected because sporocysts stay in the head-foot region of the snail for roughly 2 weeks after infection. After then, the sporocysts either migrate to the intestine of the snail (in case of susceptible snails), or some of them may veer off to the heart region (in case of resistant snails) [6, 48].

### 3.2. Bulinus Truncatus

#### 3.2.1. Effect of Diet

**Figure 4:**
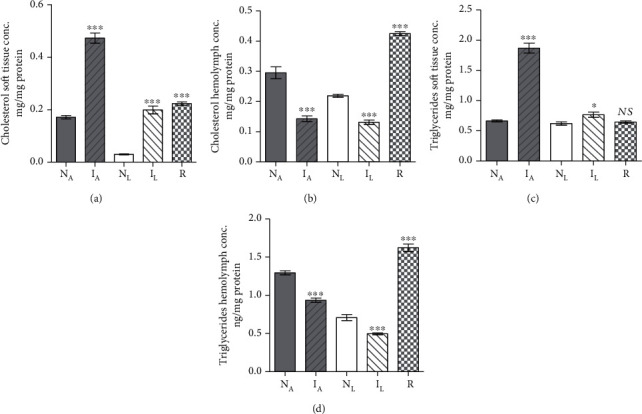
Each column represents mean soft tissue cholesterol (a), hemolymph cholesterol (b), soft tissue triglycerides (c), and hemolymph triglyceride (d) content for five *B. truncatus* snails, and vertical bars the SE about the mean. N_A_: naïve-algae fed; I_A_: infected-algae fed; N_L_: naïve-lettuce fed; I_L_: infected-lettuce fed; R: resistant snails. Statistical differences between naïve and infected or resistant same diet-fed snails are shown; ^∗∗∗^*P* < 0.001, ^∗^*P* < 0.05. NS: not significant.

**Figure 5:**
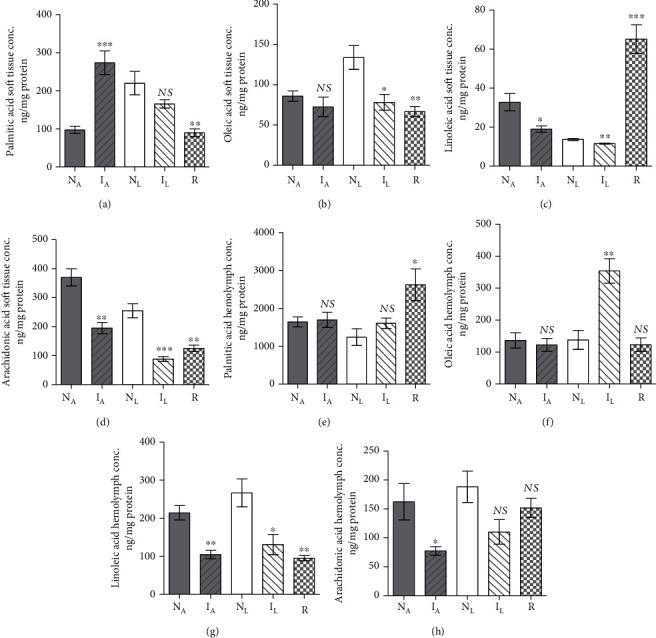
Each column represents mean soft tissue palmitic (a), oleic (b), linoleic (c), and arachidonic (d) acid and hemolymph palmitic (e), oleic (f), linoleic (g), and arachidonic (h) acid content for five *B. truncatus* snails, and vertical bars the SE about the mean. N_A_: naïve-algae fed; I_A_: infected-algae fed; N_L_: naïve-lettuce fed; I_L_: infected-lettuce fed; R: resistant snails (lettuce fed). Statistical differences between naïve and infected or resistant same diet-fed snails are shown; ^∗∗∗^*P* < 0.001, ^∗∗^0.001 < *P* < 0.005, ^∗^*P* < 0.05. NS: not significant.

Two experiments were performed in tandem. In each, five healthy snails maintained in parallel in identical conditions, except for lettuce and algae diet, were assessed for soft tissue and hemolymph protein and lipids content on an individual snail basis. The statistical analyses of results revealed that lettuce diet elicited significant (*P* < 0.005) decrease in hemolymph protein (Supplementary Tables 4, 5), soft tissue cholesterol, and triglyceride content as compared to algae-fed snails (Figures 4(a)–4(d)) and increase in soft tissue palmitic and oleic acid (Figures 5(a) and 5(b)), perhaps providing an explanation for detection of resistant snails only among those on lettuce diet. There was no otherwise remarkable effect of algae versus lettuce diet (Figure 5).

#### 3.2.2. Effect of Infection in Algae-Fed Snails

In two independent experiments, five *B. truncatus* snails maintained on algae diet were subjected to infection with *S. haematobium* miracidia and assayed, 40 days later, for soft tissue and hemolymph protein and lipids content in parallel with entirely naïve snails. Infection failed to alter the snail soft tissue protein content (Supplementary Table 4) but elicited highly significant (*P* < 0.001) increase in the hemolymph protein content (Supplementary Table 5). Changes observed after 40 days *S*. *haematobium* infection regarding cholesterol and triglyceride parameters were significant (*P* < 0.005) increase in soft tissue accompanied with decline in hemolymph (Figures 4(a)–4(d)). The most salient findings associated with *S*. *haematobium* infection were variable changes in palmitic acid levels in soft tissues and hemolymph (Figures 5(a) and 5(e)) and highly significant (up to *P* < 0.0001) decrease in soft tissue and hemolymph linoleic and arachidonic acid levels compared with naïve snails (Figure 5).

#### 3.2.3. Effect of Infection in Lettuce-Fed Snails

Snail infection with *S. haematobium* larvae for 40 days led to significantly (*P* < 0.0001) higher soft tissue and hemolymph protein content as compared to naïve snails (Supplementary Tables 4, 5). As for algae-fed snails, 40 days *S. haematobium* infection induced highly significant (*P* < 0.0001) increase in soft tissue and decline in hemolymph cholesterol and triglyceride levels (Figures 4(a)–4(d)) and variable changes in palmitic acid content (Figures 5(a) and 5(e)). Additionally, infection of lettuce-fed *B. truncatus* infection was associated with highly significant (up to *P* < 0.0001) decrease in soft tissue and hemolymph linoleic and arachidonic acid levels, when compared to naïve snails (Figure 5).

#### 3.2.4. Resistance versus Susceptibility Parameters

Laboratory bred naïve *B. truncatus* snails were maintained on algae or lettuce diet and unexposed to any infection. A series of snails were exposed twice to infection with 5-10 of *S. haematobium* miracidia, with one-month interval. The snails, which shed cercariae either after the first or the second experimental infection, were labelled susceptible. Resistant snails were found uniquely among the lettuce-fed hosts. All three groups (naïve, susceptible, and resistant) were assessed for soft tissue and hemolymph protein and lipids content on an individual snail basis. Snail resistance to infection with *S. haematobium* miracidia was associated with highly significant (*P* < 0.001) increase in soft tissue and hemolymph protein content (Supplementary Tables 4, 5) and cholesterol level in soft tissues and hemolymph (Figures 4(a) and 4(b)). Additionally, snail resistance versus susceptibility to infection was associated with variable changes in soft tissue and hemolymph palmitic acid content (Figures 5(a) and 5(e)). In sharp contrast to susceptible snails, soft tissues, but not hemolymph, of resistant snails displayed highly significant (*P* < 0.0001) increase in the levels of linoleic acid (Figures 5(c) and 5(g)) and no decrease in the levels of hemolymph oleic and arachidonic acid (Figures 5(f) and 5(h)). Snail resistance to support *S. haematobium* development remarkably correlated with the inability to decrease the soft tissues content of linoleic acid and hemolymph content of oleic and arachidonic acids to levels that are not lethal to the larvae.

#### 3.2.5. Histologic Examination

**Figure 6:**
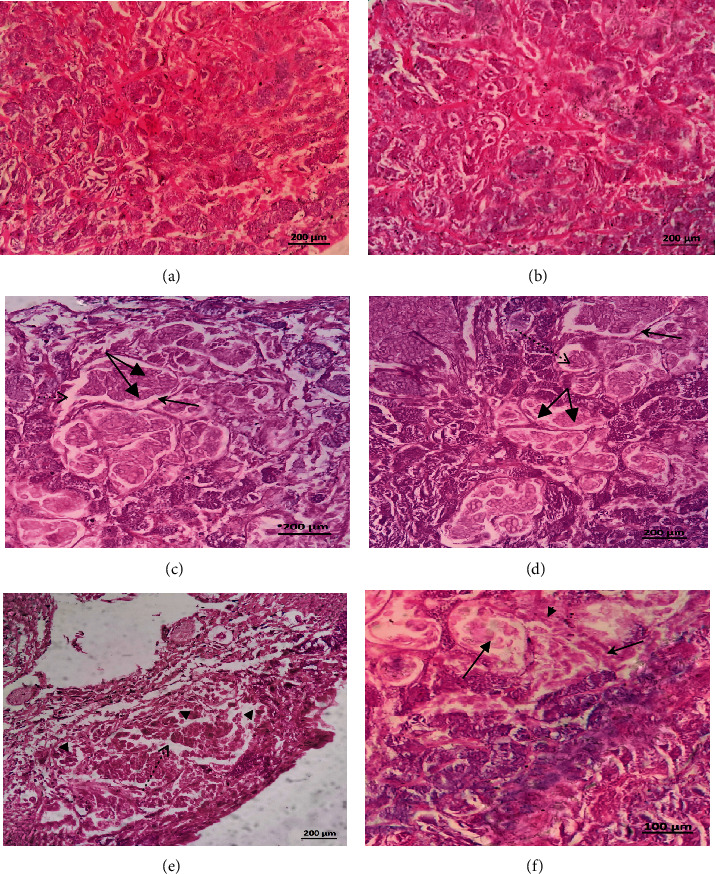
Histological transverse sections showed normal cephalopodal tissue structure of naïve *B. truncatus* snails maintained on algae or lettuce, respectively (a, b). (c, d) are of infected snails showing live mother sporocysts with normal development pattern; some of them contain single sporocyst cluster (arrowhead), and others contain multiple sporocyst clusters (twin thick arrows). All sporocysts contain viable germinal cells with nucleoli; the vacant space (dashed arrow) between the sporocyst and the tegument of the mother sporocyst also appears normal. Regarding resistant snails, cephalopodal tissue shows the sporocysts losing their characteristic cluster-like shape and dissolution of the surrounding tegument (dashed arrow), leading to dispersion of germinal cells (arrowhead) (e). The mantle collar region displays sporocycts have lost their cluster-like shape, and no viable germ cells are observed (thick arrow). Note, the negligible presence of hemocytes (thin arrow) and fibroblasts lamination (arrowhead) surrounding the sporocysts (f).

Histological transverse sections showed normal cephalopodal tissue structure of naïve *B. truncatus* snails maintained on algae or lettuce, respectively (Figures 6(a) and 6(b)). Figures 6(c) and 6(d) are of susceptible snails showing live mother sporocysts with normal development pattern. Regarding resistant snails, sporocysts showed loss of their characteristic cluster-like shape, dissolution of the surrounding tegument (dashed arrow), and dispersion of germinal cells (Figure 6(e)). The number of hemocytes and fibroblasts lamination surrounding the sporocysts appeared negligible (Figure 6(f)).

## 4. Discussion

Before exploring the possible effect of protein and lipids content on the susceptibility/resistance of snails to schistosome infection, it was essential to examine the influence of diet. Compared with lettuce, *B. alexandrina* and *B. truncatus* algae feeding led to increase in hemolymph triglycerides content, similarly to hen yolk eggs in the *Biomphalaria glabrata* model [52] and considerable increase in cholesterol content in soft tissue and hemolymph, while hen egg yolk led to *B glabrata* cholesterol increase in soft tissue but not hemolymph [53]. Hen yolk egg plus lettuce or lettuce supplemented with tetramine diet also elicited variable changes in cholesterol and triglycerides content of *B. glabrata* soft tissue [54]. Feeding *B. alexandrina* snails with algae versus lettuce led to minor increase in the palmitic and oleic acid content in soft tissue but did not affect the levels of proteins and linoleic and arachidonic acid in soft tissue and hemolymph. Similarly, lettuce and hen yolk egg diet variably affected the levels of palmitic, oleic, linoleic, arachidonic, and other fatty acids of whole *B. glabrata* snail body [55]. The data together indicate that diet impact on lipid content and distribution in the different body compartments varies with the diet composition and snail species. This conclusion should be taken into consideration regarding the feeding strategies of parasite vectors and edible snails.

Successful infection of algae- or lettuce-fed *B. alexandrina* and *B. truncatus* with schistosome miracidia similarly elicited dramatic increase in soft tissue cholesterol and triglycerides content versus decrease in hemolymph. Likewise, *S. mansoni* infection of *B. glabrata* led to considerable changes in soft tissue amounts of cholesterol and triglycerides [56, 57]. The results of the present study corroborate the report of lipid content reduction (80%) after 60 days infection of *B. alexandrina* with *S. mansoni*, with lipids measured involving triacylglycerols and free sterols as well as free fatty acids [58]. Indeed, the most striking and consistent parameter change in our snails study was the association of susceptibility to schistosome infection with highly significant (up to *P* < 0.0001) decrease in soft tissue and hemolymph oleic, linoleic, and arachidonic acid content. These results suggest that *S. mansoni* and *S. haematobium* intramolluscan development is successful provided low levels of mono- and especially polyunsaturated fatty acids in soft tissue. In support, resistance of *B*. *alexandrina* and *B. Bulinus* snails to schistosome infection appeared to directly correlate with inability to decrease the soft tissue content of oleic, linoleic, and arachidonic acid. Polyunsaturated acids, especially arachidonic acid, appeared thus to affect the development of schistosomes in both the intermediate snail and final mammalian [36–42] host. Successful development of S. *mansoni* had to be accompanied by more than 50% decrease of *B. alexandrina* soft tissue mono- and polyunsaturated fatty acids. *Schistosoma haematobium*, known for their higher sensitivity to PUFAs [40–44], would have never approached *Bulinus truncatus* if it carried the PUFA levels of *B. alexandrina.* Of note, invading schistosomes were indifferent to the levels of palmitic acid in snail soft tissue and hemolymph, while decrease in PUFAS levels strongly support theit function as endogenous antimicrobial molecules [59].

Our results document for the first time a limited, but significant, role of diet in snails innate immune responses, as snail resistant to second infection was 0% in algae versus approximately 10% in lettuce-fed snails. Additionally, our findings are in accord with both the “resistance” and the “matching” hypotheses [21, 24, 25]. Upon invasion, parasites are able to immediately dampen the snail PUFA synthesis pathways, survive, and reproduce. Parasite elimination occurs when the schistosome-host interaction fails to elicit decline in the soft tissue PUFA content, and on the contrary, leads to its increase. It might be useful to determine the mechanism(s) via which invading miracidia manipulate PUFA biosynthetic machinery [60–63]. Data have been obtained documenting the ability of the schistosome to manipulate the snail genome, including the expression of specific genes [26, 64, 65]. Yet, numerous transcripts critical to *S. mansoni* intra-*Biomphalaria* development are not identified as yet, and details on how considerable infection hurts the snail host metabolism are lacking [63, 65]. The present findings suggested that snail susceptibility/resistance to schistosomes is not uniquely dependent on hemocyte abundance and activity [26–33, 64]. Hemocyte migration and function may be easily impaired by several sporocyst-derived factors, among which antioxidant and immune-modulator molecules, and proteolytic enzymes, notably the metalloproteinase, SmLeish [66–70]. Fortunately, the parasites also fail to develop due to a plethora of intramolluscan immune factors and miracidia deterrents and biochemically unsuitable determinants [21, 26–34, 64, 71–73]. Moreover, PUFA level is a target that could be manipulated via diet to prevent snail infection, using PUFA (notably arachidonic acid) synthesizing algae, fungi, and plants [74]. It is, however, necessary to investigate whether such approach leads to snail over population or affects aquaculture fauna populations.

## 5. Conclusions

Laboratory-bred, algae-, or lettuce-fed *Biomphalaria alexandrina* and *Bulinus truncatus* adult snails were exposed to miracidia of long-term laboratory maintained Egyptian strains of *Schistosoma mansoni* and *S. haematobium*, respectively. Less than 50% of exposed snails showed patent infection 30 and 40 days after exposure, respectively, while around 10% of lettuce-fed snails resisted not once but twice infection with miracidia. Over two experiments, biochemical investigations of snails from each group revealed consistent and reproducible correlation between successful infection and highly significant decrease in oleic, linoleic, and arachidonic acid content in soft tissue and hemolymph, suggesting susceptibility of schistosome larvae to critical PUFA concentration. In support, the snails that resisted twice infection had soft tissue PUFA levels as high as unexposed naïve snails. The data together led to recommending manipulating the schistosome intermediate snail host diet towards excessive accumulation of PUFAs in soft tissue and hemolymph. The aim is to interfere with intrasnail schistosome development and transmission of infection, taking into consideration the effects of that approach on snail and other aquaculture fauna maturation and reproduction.

## Data Availability

All data are shown in tables, figures, and supplementary materials.

## Supplementary Materials

Supplementary Materials Figure S1: Multiple Reaction Monitoring (MRM) chromatograms of DHA-the internal standard- (A), palmitic (B), oleic (C), linoleic (D) and arachidonic (E) fatty acids, using the optimized method conditions at their selective transitions (Table S1). Table S1A: method validation parameters for palmitic acid: precision, accuracy, and recovery. ^∗^a: intraday precision, b: interday precision; Table 1B: method validation parameters for oleic acid: precision, accuracy, and recovery. ^∗^a: intraday precision, b: interday precision; Table 1C: method validation parameters for linoleic acid: precision, accuracy, and recovery. ^∗^a: intraday precision, b: interday precision; Table 1D: method validation parameters for arachidonic acid: precision, accuracy, and recovery. ^∗^a: intraday precision, b: interday precision. Table S2: soft tissue protein content of *Biomphalaria alexandrina* snails. Table S3: hemolymph protein content of *Biomphalaria alexandrina* snails. Table S4: soft tissue protein content of *Bulinus truncatus* snails. Table S5: hemolymph protein content of *Bulinus truncatus* snails.
