# Supplementary material for: Resistance of Biomphalaria alexandrina to Schistosoma mansoni and Bulinus truncatus to Schistosoma haematobium Correlates with Unsaturated Fatty Acid Levels in the Snail Soft Tissue
Source: J Parasitol Res. 2020 Nov 1;2020:8852243. doi: 10.1155/2020/8852243 (PMC7652611; doi:10.1155/2020/8852243)
Supplement: Supplementary Materials — Figure S1: Multiple Reaction Monitoring (MRM) chromatograms of DHA-the internal standard- (A), palmitic (B), oleic (C), linoleic (D) and arachidonic (E) fatty acids, using the optimized method conditions at their selective transitions (Table S1). Table S1A: method validation parameters for palmitic acid: precision, accuracy, and recovery. ∗a: intraday precision, b: interday precision; Table 1B: method validation parameters for oleic acid: precision, accuracy, and recovery. ∗a: intraday precision, b: interday precision; Table 1C: method validation parameters for linoleic acid: precision, accuracy, and recovery. ∗a: intraday precision, b: interday precision; Table 1D: method validation parameters for arachidonic acid: precision, accuracy, and recovery. ∗a: intraday precision, b: interday precision. Table S2: soft tissue protein content of Biomphalaria alexandrina snails. Table S3: hemolymph protein content of Biomphalaria alexandrina snails. Table S4: soft tissue protein content of Bulinus truncatus snails. Table S5: hemolymph protein content of Bulinus truncatus snails. [file 8852243.f1.docx]

Supplementary TABLE 1:

| Labeled  Concentrations  (ng/ml) | Measured Concentrations (ng/ml) | | | | | | Mean | Precision  CV% | Accuracy  Bias% | Recovery  R% |
| --- | --- | --- | --- | --- | --- | --- | --- | --- | --- | --- |
|  | 1 | 2 | 3 | 4 | 4ꞌꞌ | 4ꞌꞌꞌ |  |  |  |  |
| 50 | 49.484 | 48.869 | 48.298 | 47.814 | 46.433 | 56.746 | 50.774 | 1.484^a^, 8.271^b^ | 1.548 | 101.548 |
| 500 | 486.209 | 485.436 | 493.454 | 481.751 | 487.100 | 486.058 | 486.668 | 1.006^a^, 0.585^b^ | -2.666 | 97.334 |
| 2000 | 2017.088 | 2011.093 | 2012.929 | 2023.605 | 1996.846 | 1989.562 | 2008.521 | 0.275^a^, 0.895^b^ | 0.426 | 100.426 |
| 6000 | 5991.532 | 5993.304 | 5999.812 | 5988.600 | 5971.346 | 6007.905 | 5992.083 | 0.079^a^, 0.305^b^ | -0.132 | 99.868 |
| 10000 | 10019.299 | 10006.686 | 10035.852 | 10213.101 | 10047.482 | 10233.688 | 10092.685 | 0.963^a^, 1.004^b^ | 0.927 | 100.927 |
| 18000 | 18011.560 | 18047.940 | 17971.181 | 17318.407 | 17885.816 | 17637.183 | 17812.014 | 1.947^a^, 1.615^b^ | -1.044 | 98.956 |

TABLE S1A: Method validation parameters for palmitic acid: precision, accuracy and recovery. *a: intra-day precision, b: inter-day precision.

| Labeled  Concentrations  (ng/ml) | Measured Concentrations (ng/ml) | | | | | | Mean | Precision  CV% | Accuracy  Bias% | Recovery  R% |
| --- | --- | --- | --- | --- | --- | --- | --- | --- | --- | --- |
|  | 1 | 2 | 3 | 4 | 4ꞌꞌ | 4ꞌꞌꞌ |  |  |  |  |
| 50 | 51.829 | 54.947 | 45.183 | 46.246 | 47.943 | 46.876 | 48.837 | 9.055^a^,6.629^b^ | -3.659 | 96.341 |
| 500 | 495.336 | 497.413 | 502.289 | 494.085 | 498.854 | 494.938 | 497.153 | 0.726^a^,0.513^b^ | -0.569 | 99.431 |
| 2000 | 2000.283 | 1998.816 | 2013.369 | 2006.981 | 1999.212 | 2003.426 | 2003.681 | 0.334^a^,0.194^b^ | 0.184 | 100.184 |
| 6000 | 5996.401 | 5990.675 | 5997.542 | 5992.043 | 5986.960 | 5996.211 | 5993.305 | 0.055^a^,0.077^b^ | -0.112 | 99.888 |
| 10000 | 10050.968 | 10033.598 | 10010.002 | 9981.410 | 9978.151 | 9981.106 | 10005.872 | 0.301^a^,0.018^b^ | 0.059 | 100.059 |
| 18000 | 17992.829 | 17922.820 | 17947.127 | 17900.733 | 17957.765 | 17945.933 | 17944.535 | 0.220^a^,0.168^b^ | -0.308 | 99.692 |

TABLE S1B: Method validation parameters for oleic acid: precision, accuracy and recovery. *a: intra-day precision, b: inter-day precision.

TABLE S1C: Method validation parameters for linoleic acid: precision, accuracy and recovery. *a: intra-day precision, b: inter-day precision.

| Labeled  Concentrations  (ng/ml) | Measured Concentrations (ng/ml) | | | | | | Mean | Precision  CV% | Accuracy  Bias% | Recovery  R% |
| --- | --- | --- | --- | --- | --- | --- | --- | --- | --- | --- |
|  | 1 | 2 | 3 | 4 | 4ꞌꞌ | 4ꞌꞌꞌ |  |  |  |  |
| 50 | 48.62 | 54.929 | 48.153 | 47.266 | 49.132 | 53.740 | 49.743 | 7.042^a^,6.659^b^ | 0.615 | 100.615 |
| 500 | 496.24 | 526.030 | 485.873 | 505.077 | 500.210 | 506.261 | 503.305 | 3.390^a^,0.636^b^ | 0.656 | 100.656 |
| 2000 | 2013.19 | 1860.069 | 2066.488 | 1950.037 | 2006.725 | 1979.530 | 1972.445 | 4.500^a^,1.433^b^ | -1.033 | 98.967 |
| 6000 | 6001.23 | 5963.794 | 6004.404 | 5972.106 | 6003.944 | 5995.468 | 5985.384 | 0.342^a^,0.275^b^ | -0.164 | 99.836 |
| 10000 | 10000.83 | 9588.758 | 9966.837 | 10076.586 | 9545.006 | 9967.590 | 9908.252 | 2.199^a^,2.847^b^ | -1.424 | 98.576 |
| 18000 | 17996.30 | 17966.163 | 17926.746 | 18035.366 | 18039.029 | 18103.193 | 17981.143 | 0.256^a^,0.211^b^ | 0.062 | 100.062 |

| Labeled  Concentrations  (ng/ml) | Measured Concentrations (ng/ml) | | | | | | Mean | Precision  CV% | Accuracy  Bias% | Recovery  R% |
| --- | --- | --- | --- | --- | --- | --- | --- | --- | --- | --- |
|  | 1 | 2 | 3 | 4 | 4ꞌꞌ | 4ꞌꞌꞌ |  |  |  |  |
| 50 | 53.329 | 53.088 | 55.175 | 45.029 | 52.738 | 50.945 | 51.717 | 8.741a,8.139b | 3.435 | 103.435 |
| 500 | 498.290 | 513.950 | 505.822 | 504.810 | 506.601 | 511.676 | 506.858 | 1.270a,0.702b | 1.372 | 101.372 |
| 2000 | 2002.176 | 2011.215 | 2034.534 | 2039.994 | 2041.213 | 1988.993 | 2019.688 | 0.899a,1.473b | 0.984 | 100.984 |
| 6000 | 6000.202 | 6021.095 | 6033.629 | 6037.863 | 6036.855 | 6030.555 | 6026.700 | 0.281a,0.066b | 0.445 | 100.445 |
| 10000 | 9967.864 | 9763.654 | 9969.582 | 9837.969 | 10208.290 | 9781.533 | 9921.482 | 1.028a,2.332b | -0.785 | 99.215 |
| 18000 | 18010.204 | 18002.494 | 18040.409 | 18076.101 | 18027.532 | 18045.486 | 18033.704 | 0.186a,0.136b | 0.187 | 100.187 |

TABLE S1D: Method validation parameters for arachidonic acid: precision, accuracy and recovery. *a: intra-day precision, b: inter-day precision.

| 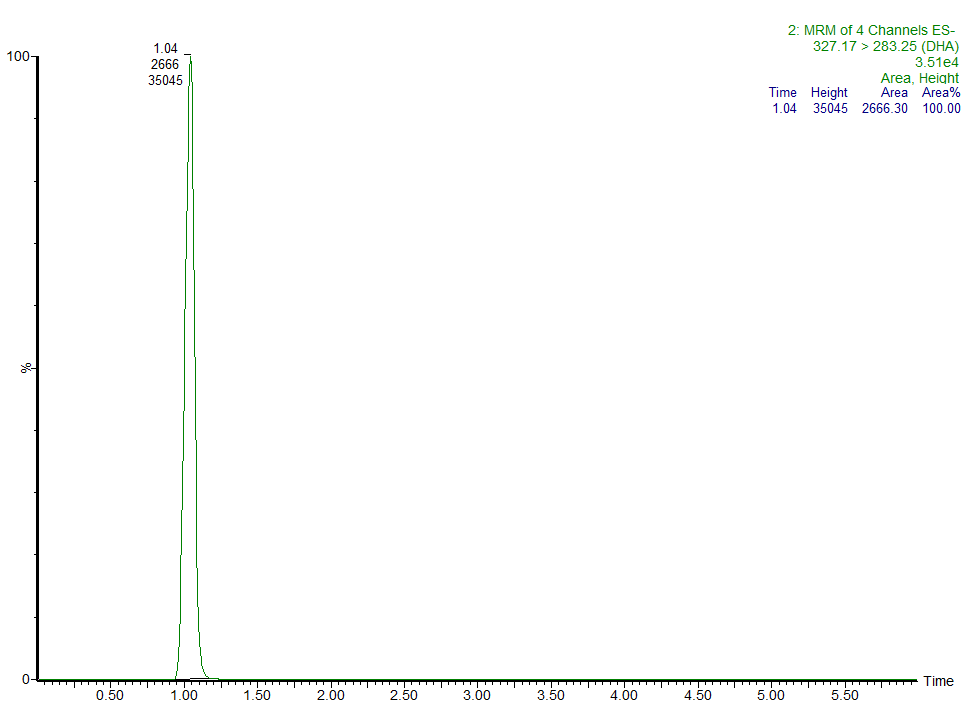  **A** |
| --- |
| 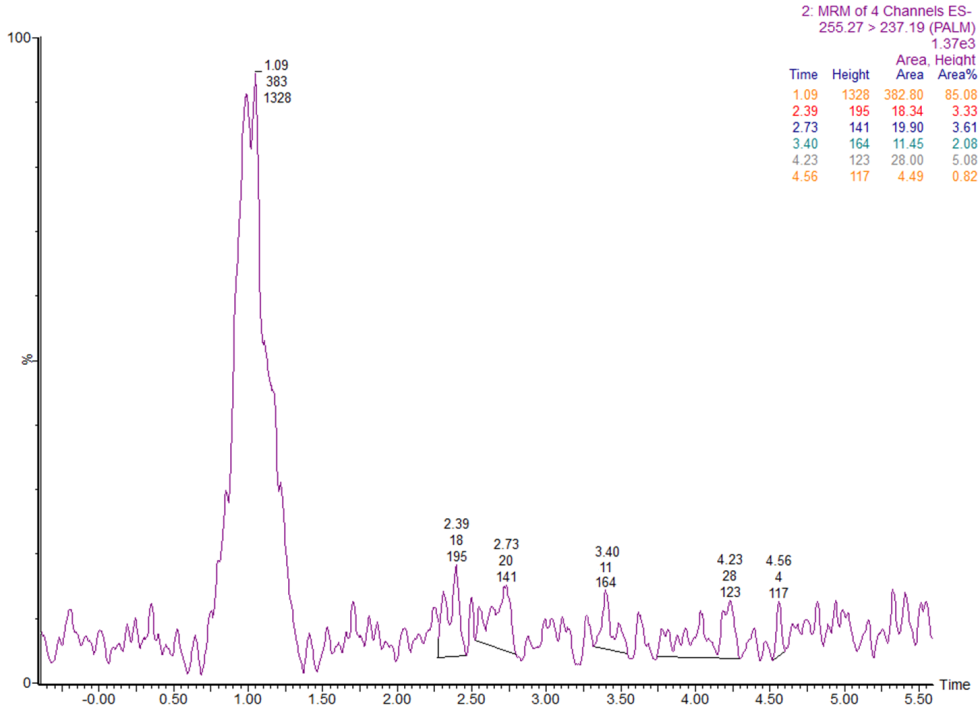  **B** |

| **C** |
| --- |
| 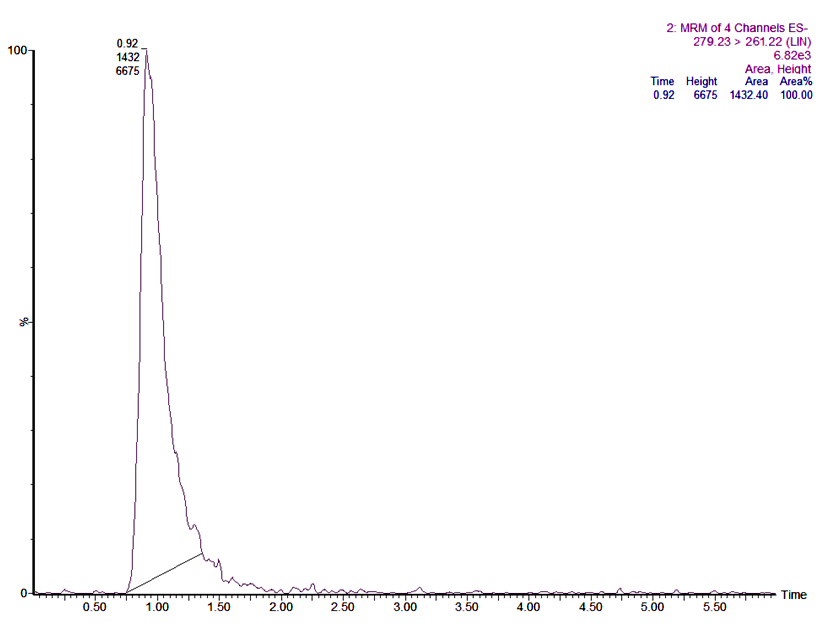  **D** |


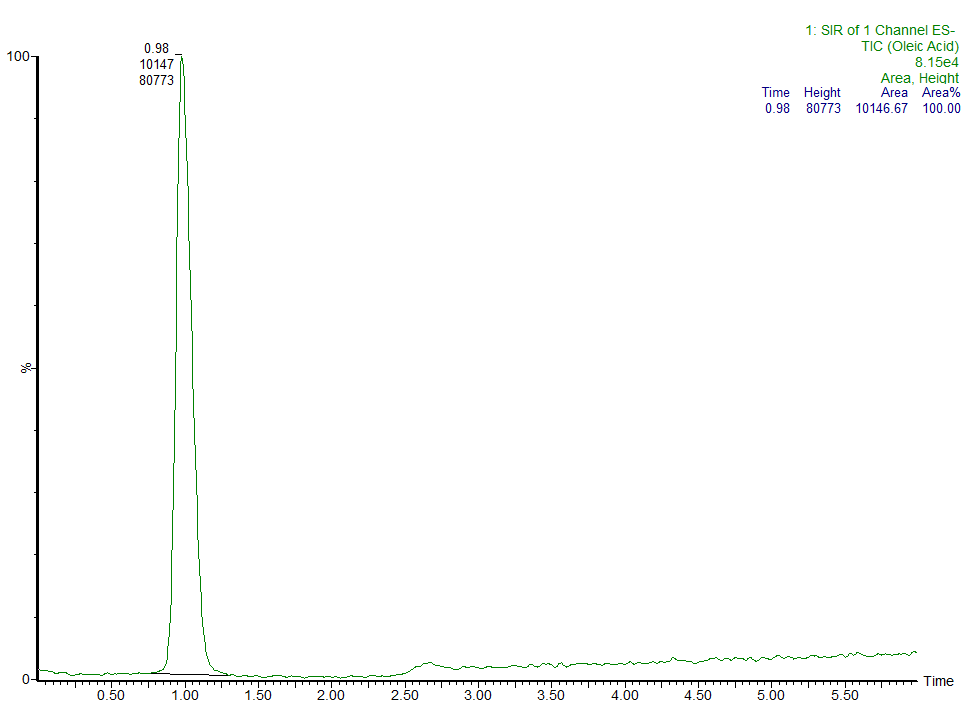


| **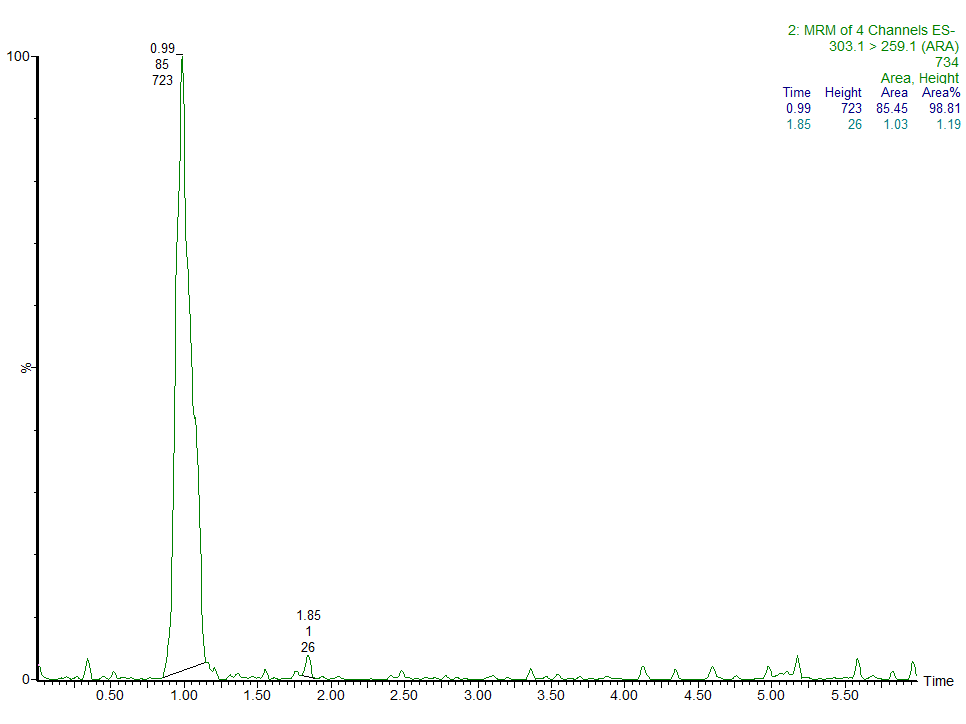**  **E** |
| --- |

Supplementary FIGURE 1: Multiple Reaction Monitoring (MRM) chromatograms of DHA-the internal standard- (A), Palmitic (B), Oleic (C), Linoleic (D) and Arachidonic (E) fatty acids, using the optimized method conditions at their selective transitions (Table 1).

Supplementary TABLE 2: Soft tissue protein content of *Biomphalaria alexandrina* snails.

| Sample Number | | Soft tissue protein content (mg/ml) | | | | |
| --- | --- | --- | --- | --- | --- | --- |
|  |  | N_A_ | I_A_ | N_L_ | I_L_ | R |
| 1 | | 1.908 | 2.026 | 1.911 | 1.948 | 1.556 |
| 2 | | 1.959 | 1.865 | 1.930 | 1.878 | 1.857 |
| 3 | | 2.010 | 2.131 | 1.892 | 1.938 | 1.809 |
| 4 | | 1.801 | 2.069 | 2.037 | 1.854 | 1.803 |
| 5 | | 2.016 | 2.040 | 1.873 | 1.959 | 1.639 |
| Mean | | 1.939 | 2.026 | 1.928 | 1.916 | 1.733 |
| SEM | | 0.040 | 0.044 | 0.029 | 0.021 | 0.058 |
| *P* value | N_A_ vs N_L_ | 0.84001 | | | | |
|  | I_A_ vs I_L_ | 0.05293 | | | | |
|  | N_A_ vs I_A_ | 0.17820 | | | | |
|  | N_L_ vs I_L_ | 0.72515 | | | | |
|  | R vs N_L_ | 0.01597 | | | | |
|  | R vs I_L_ | 0.01734 | | | | |

Näive-algae fed, N_A_, infected-algae fed, I_A_, näive-lettuce fed, N_L_, infected-lettuce fed, I_L_, resistant snails, R.

Supplementary TABLE 3: Hemolymph protein content of *Biomphalaria alexandrina* snails.

| Sample Number | | Hemolymph protein content (mg/ml) | | | | |
| --- | --- | --- | --- | --- | --- | --- |
|  |  | N_A_ | I_A_ | N_L_ | I_L_ | R |
| 1 | | 2.913 | 3.056 | 2.626 | 2.795 | 2.274 |
| 2 | | 2.717 | 2.752 | 2.631 | 2.725 | 2.158 |
| 3 | | 3.136 | 2.757 | 2.634 | 2.760 | 2.182 |
| 4 | | 2.876 | 3.010 | 2.572 | 2.975 | 2.150 |
| 5 | | 2.948 | 2.671 | 2.644 | 2.755 | 2.120 |
| Mean | | 2.918 | 2.849 | 2.621 | 2.802 | 2.177 |
| SEM | | 0.067 | 0.077 | 0.013 | 0.045 | 0.026 |
| *P* value | N_A_ vs N_L_ | 0.00252 | | | | |
|  | I_A_ vs I_L_ | 0.60896 | | | | |
|  | N_A_ vs I_A_ | 0.51966 | | | | |
|  | N_L_ vs I_L_ | 0.00462 | | | | |
|  | R vs N_L_ | 3.315E-07 | | | | |
|  | R vs I_L_ | 2.038E-06 | | | | |

Näive-algae fed, N_A_, infected-algae fed, I_A_, näive-lettuce fed, N_L_, infected-lettuce fed, I_L_, resistant snails, R.

Supplementary TABLE 4: Soft tissue protein content of *Bulinus truncatus* snails.

| Sample Number | | Soft Tissue | | | | |
| --- | --- | --- | --- | --- | --- | --- |
|  |  | N_A_ | I_A_ | N_L_ | I_L_ | R |
| 1 | | 1.768 | 1.919 | 1.330 | 1.948 | 2.513 |
| 2 | | 2.013 | 1.857 | 1.376 | 1.903 | 2.653 |
| 3 | | 1.938 | 1.655 | 1.521 | 1.919 | 2.424 |
| 4 | | 1.790 | 1.954 | 1.422 | 2.067 | 2.760 |
| 5 | | 1.827 | 1.924 | 1.298 | 2.102 | 2.706 |
| Mean | | 1.867 | 1.862 | 1.389 | 1.988 | 2.611 |
| SEM | | 0.047 | 0.054 | 0.039 | 0.040 | 0.062 |
| *P* value | N_A_ vs N_L_ | 0.00049 | | | | |
|  | I_A_ vs I_L_ | 0.09907 | | | | |
|  | N_A_ vs I_A_ | 0.94622 | | | | |
|  | N_L_ vs I_L_ | 0.00053 | | | | |
|  | R vs N_L_ | 0.00017 | | | | |
|  | R vs I_L_ | 0.00031 | | | | |

Näive-algae fed, N_A_, infected-algae fed, I_A_, näive-lettuce fed, N_L_, infected-lettuce fed, I_L_, resistant snails, R.

Supplementary TABLE 5**:** Hemolymph protein content of *Bulinus truncatus* snails.

| Sample Number | | Soft Tissue | | | | |
| --- | --- | --- | --- | --- | --- | --- |
|  |  | N_A_ | I_A_ | N_L_ | I_L_ | R |
| 1 | | 2.677 | 3.198 | 2.607 | 3.276 | 2.932 |
| 2 | | 2.682 | 3.126 | 2.618 | 3.144 | 2.951 |
| 3 | | 2.881 | 3.204 | 2.795 | 3.233 | 3.109 |
| 4 | | 2.833 | 3.308 | 2.846 | 3.279 | 2.892 |
| 5 | | 2.951 | 3.303 | 2.857 | 3.247 | 2.943 |
| Mean | | 2.805 | 3.228 | 2.744 | 3.236 | 2.965 |
| SEM | | 0.054 | 0.035 | 0.055 | 0.024 | 0.037 |
| *P* value | N_A_ vs N_L_ | 0.45921 | | | | |
|  | I_A_ vs I_L_ | 0.85401 | | | | |
|  | N_A_ vs I_A_ | 0.00018 | | | | |
|  | N_L_ vs I_L_ | 0.00037 | | | | |
|  | R vs N_L_ | 0.01053 | | | | |
|  | R vs I_L_ | 0.00036 | | | | |

Näive-algae fed, N_A_, infected-algae fed, I_A_, näive-lettuce fed, N_L_, infected-lettuce fed, I_L_, resistant snails, R.
